# Osteopathy may decrease obstructive apnea in infants: a pilot study

**DOI:** 10.1186/1750-4732-2-8

**Published:** 2008-07-19

**Authors:** Yvan Vandenplas, Etienne Denayer, Thierry Vandenbossche, Luc Vermet, Bruno Hauser, Jean DeSchepper, Agnes Engelen

**Affiliations:** 1 Department of Pediatrics, Universitair Ziekenhuis Brussel Kinderen, Brussels, Belgium

## Abstract

**Background:**

Obstructive apnea is a sleep disorder characterized by pauses in breathing during sleep: breathing is interrupted by a physical block to airflow despite effort. The purpose of this study was to test if osteopathy could influence the incidence of obstructive apnea during sleep in infants.

**Methods:**

Thirty-four healthy infants (age: 1.5–4.0 months) were recruited and randomized in two groups; six infants dropped out. The osteopathy treatment group (n = 15 infants) received 2 osteopathic treatments in a period of 2 weeks and a control group (n = 13 infants) received 2 non-specific treatments in the same period of time. The main outcome measure was the change in the number of obstructive apneas measured during an 8-hour polysomnographic recording before and after the two treatment sessions.

**Results:**

The results of the second polysomnographic recordings showed a significant decrease in the number of obstructive apneas in the osteopathy group (p = 0.01, Wilcoxon test), in comparison to the control group showing only a trend suggesting a gradual physiologic decrease of obstructive apneas. However, the difference in the decline of obstructive apneas between the groups after treatment was not significant (p = 0.43).

**Conclusion:**

Osteopathy may have a positive influence on the incidence of obstructive apneas during sleep in infants with a previous history of obstructive apneas as measured by polysomnography. Additional research in this area appears warranted.

## Introduction

The aim of the study was to measure the impact of osteopathic treatment on the incidence of obstructive apnea in infants. Obstructive apnea is a pathologic finding, and is in some infants related to a risk for sudden infant death. The infants were recruited from a broad population of children younger than 4 months who tested positive according to a polysomnographic test, which is known to be a reliable and reproducible diagnostic tool for obstructive apnea [1].

## Methods

Infants presenting with episodes of pallor, that were severe enough to alarm their parents or other caregivers, were submitted to an 8-hour polysomnographic recording measuring heart rate, respiratory rate and movements, electroencephalogram, oxygen saturation, electromyography, eye movements (Morpheus^®^, Medatec, Brussels, Belgium). All registrations were read out by one expert (ED). The polysomnographic examination was interpreted according to the criteria of the Belgian National Institute for Insurance and Invalidity (R.I.Z.I.V.), based on published and accepted scientific criteria. These criteria state that an 8-hour polysomnographic recording is abnormal if one of the following criteria is met: (a) more than one central apnea of more than 20 seconds with a desaturation (SaO_2 _< 88%); (b) bradycardia of less than 60 beats per minute; (c) more than 3 obstructive apneas lasting more than 3 seconds [2]. If the recording was "positive", a home cardiorespiratory monitoring was started.

Participation in this study was proposed to all parents of infants in whom the 8-hour polysomnographic recording revealed more than 3 obstructive apneas [2]. Overall, participation was proposed to the parents of 142 infants meeting the inclusion criteria.

The trial consisted of an evaluator- and patient-blinded methodology, evaluating the effect of two sessions of osteopathic treatment or non-specific treatment in a period of 2 weeks on the number of obstructive apneas recorded with a second polysomnography. We developed an evaluator-blinded and patient-blinded, placebo-controlled design to ensure that: (a) only the osteopath knew which infant had been given a genuine osteopathic treatment and which infant had not; (b) the parents and the other researchers did not know which infant was treated with osteopathy and which infant was not; (c) the main outcome of the study, the result of the polysomnographic recording, was interpreted by a blinded investigator. The treatment sessions were scheduled during the weekend. The osteopath giving the treatment and the investigator reading the polysomnographic recordings never had any direct contact. After the parents gave their consent for participation in the study, infants were randomized. The osteopathic and non-specific treatments were all given by one osteopath with experience of over 30 years (AE). In the non-specific treatment group, gentle mobilisations (flexion, extension and rotation) of the extremities were performed in a standardised sequence. After performing an osteopathic diagnostic evaluation, the infants in the osteopathic treatment group were mainly treated with functional techniques for the specific dysfunctions found at that visit. In this group a "black box" design was chosen to meet the individuality of the child and the treatment principles of osteopathy. In both groups, each session took about 30 minutes. The first treatment was given one week after the first polysomnography, the second treatment was given two weeks after the first treatment. The second polysomnographic recording was performed 4 weeks after the first polysomnography.

The primary outcome was the result of the second polysomnographic recording. The secondary outcome was the number of infants whose polysomnographic recording had normalised.

The study was approved by the local Ethical Committee. Since the number of apneas was not normally distributed, variation from baseline was analyzed using the non-parametric Wilcoxon rank sum test and the differences between the groups by the Mann-Whitney *U *test. The Mann-Whitney U test is used for independent samples, while the Wilcoxon Signed Rank test is used for paired samples or when repeated measures techniques are used. The difference in number of patients with normalized polysomnography between the intervention and control group was analysed by the Fisher exact test. The level of significance was set at p < 0.05.

## Results

The initial study design was planned to include 40 infants during a one-year interval. However, after one year, only 34 parents (out of 142 eligible candidates) had given their written consent for participation (Table 1). Five participants dropped out because of: illness of the infant during the week in which the second polysomnographic recording was planned (n = 2), refusal of the second polysomnographic recording (n = 2), and poor technical quality of the second recording (n = 1). A sixth infant was excluded because it was diagnosed with Werdnig-Hofmann myopathy at a later age (Werdnig-Hofmann myopathy is a lethal myopathy in which the respiratory muscles gradually become more paralysed, until spontaneous respiration becomes impossible).

**Table 1:** Consort statement describing subject flow

	Number of patients
Eligible for inclusion	142
Parents accepting participation	34
First polysomnography	34
Drop-outs	6
Acute disease at 2^nd ^polysomnography	2
Refusal of second polysomnography	2
Poor technical quality of the second recording	1
Werdnig-Hoffman myopathy	1
Second polysomnography	28
Osteopathy	15
Non-specific treatment	13

The results of 28 infants were analysed. At inclusion, all infants had more than three obstructive apneas during the recording; there was no infant with central apnea of longer than 20 seconds, significant bradycardia (< 60 beats/min) or significant oxygen desaturation (decrease of >10% [normal saturation is around 98%]). Because the inclusion of the 40 planned infants could not be realised and because of the drop-outs, the distribution between both groups was not equal: osteopathic treatment was applied to 15 infants (8 males and 7 females); 13 (7 males and 6 females) received non-specific treatment. The mean age of the osteopathy group was 57.5 days (range, 37–70 days; SD, 10.4 days); the mean age in the control group was 70.3 days (range, 48–118 days; SD, 18.4 days) (NS). In the osteopathy group, the mean birth weight was 3.3 kg (range, 2.4–4.1 kg; SD, 0.5 kg), and the mean birth weight in the control group was 3.0 kg (range, 1.5–3.7 kg; SD, 0.6 kg) (NS).

In the osteopathy group, 35% of the infants had an imbalance at the level of the upper thoracic zone (thoracic vertebrae 2–3). One in two children had a preference for head rotation towards their right side, denoting a high cervical or occipital dysfunction.

The second polysomnographic recording showed a significant decrease of the number of obstructive apneas in the osteopathic treatment group. The number of obstructive apneas had decreased almost twice as much in the osteopathic treatment group as in the control group (46.5% reduction versus 27.1% reduction). The decline in the number of obstructive apneas in the osteopathic treatment group was mean, 5.7; SD, 9.2 versus mean, 2.9; SD, 8.8 in the non-specific treatment group (Table 2). However, there was no statistically significant difference in the decline in the number of obstructive apneas between both groups (osteopathy versus non-specific treatment) (p = 0.43).

**Table 2:** Number of obstructive apneas recorded by polysomnography at baseline and after treatment*

	**Osteopathy**	**Non-specific treatmen**t
**Baseline**		
Median	11.0	10.0
Mean ± 1 SD	12.2 ± 8.0	10.8 ± 6.0
Range	5 – 32	5 – 23

**After treatment**		
Median	4.0	6.0
Mean ± 1 SD	6.5 ± 11.1	7.9 ± 6.9
Range	0 – 45	1 – 27

**Difference**		
Median	7.0	4.0
Mean ± 1 SD	5. 7 ± 9.2	2.9 ± 8.8
Range	-20 – 23	-21 – 17
Decrease (mean %)	46.5	27.1

P (Wilcoxon)	0.01	0.07

*Difference in number of obstructive apneas between groups after treatment was not statistically significant (p = 0.43).

In the osteopathy group, the second polysomnography was still positive (>3 obstructive apneas/8-hour recording) in 8/15 infants, whereas 9/13 control infants had a second positive polysomnography. However, the difference between the two groups was not significant (p = 0.46 for the Fisher exact test) (Table 3).

**Table 3:** Number of infants with a positive polysomnographic recording*

	**Osteopathy**	**Non-Specific Treatment**
**Baseline**	15 (100%)	13 (100%)
**Second recording**	8 (53%)	9 (69%)

* p = 0.46 (Fisher exact test).

## Discussion

To the best of our knowledge, this is the first study with or without a blinded design to measure the effect of osteopathic treatment in infants with an increased incidence of obstructive apnea. We had difficulties in convincing parents to take part in this evaluator-blinded and patient-blinded, placebo-controlled interventional trial evaluating osteopathy versus non-specific treatment. Many parents seemed to be either non-believers (and refused participation) or believers (refusing participation because of the non-specific treatment).

The literature is clear on one aspect regarding the etiology of obstructive apnea in infants: many hypotheses have been suggested but none is satisfactory. The heterogeneity of possible causes is likely to be the culprit. One hypothesis is that there is a relationship between the (lack of) maturation of the neurovegetative system and the cardiorespiratory system [3].

The diagnostic information we obtained in this research study was that 35% of the treated infants had an imbalance at the level of the upper thoracic zone (thoracic vertebrae 2–3). This zone could be in direct or indirect relation with heart, lungs, stomach (contraction of the pylorus), plexus solaris or regulation of blood pressure [4]. One in two children had a preference for head rotation towards their right side, denoting a high cervical or occipital dysfunction, as was observed by Carreiro [5]. The other children had no preferred side for head rotation. The reason for this observation (preferred head rotation to the right in half of the children) is not clear, and should be further investigated. As each osteopath has a personal approach toward each individual patient, all treatments were performed by the same, experienced osteopath to minimize inter-practitioner variability in treatment approaches. Thus, the study represents more the efficacy of that osteopath rather than the efficacy of osteopathy in general.

We conclude in this study that osteopathic treatment may have a positive influence on the reduction of the number of obstructive apneas during sleep in infants younger than 4 months. The number of obstructive apneas decreased in a statistically significant way within the osteopathy group, although there was no significant difference between groups in the decline in the number of obstructive apneas after treatment. This is, of course, a very preliminary study. However, the results suggest support for further research in this area.
